# Renewable Power Generation by Reverse Electrodialysis Using an Ion Exchange Membrane

**DOI:** 10.3390/membranes11110830

**Published:** 2021-10-28

**Authors:** Sourayon Chanda, Peichun Amy Tsai

**Affiliations:** Department of Mechanical Engineering, University of Alberta, 9211 116 St. NW, Edmonton, AB T6G 1H9, Canada; sourayon@ualberta.ca

**Keywords:** renewable energy, reverse electrodialysis, ion-exchange membrane

## Abstract

Reverse electrodialysis (RED) is a promising technology to extract sustainable salinity gradient energy. However, the RED technology has not reached its full potential due to membrane efficiency and fouling and the complex interplay between ionic flows and fluidic configurations. We investigate renewable power generation by harnessing salinity gradient energy during reverse electrodialysis using a lab-scaled fluidic cell, consisting of two reservoirs separated by a nanoporous ion exchange membrane, under various flow rates ($q_{f}$) and salt-concentration difference (Δc). The current-voltage (*I*-*V*) characteristics of the single RED unit reveals a linear dependence, similar to an electrochemical cell. The experimental results show that the change of inflow velocity has an insignificant impact on the *I*-*V* data for a wide range of flow rates explored (0.01–1 mL/min), corresponding to a low-Peclet number regime. Both the maximum RED power density ($P_{c,m}$) and open-circuit voltage ($\phi_{0}$) increase with increasing Δc. On the one hand, the RED cell’s internal resistance ($R_{c}$) empirically reveals a power-law dependence of $R_{c}\propto \Delta c^{-\alpha }$. On the other hand, the open-circuit voltage shows a logarithmic relationship of $\phi_{0}=B\ln\Delta c+\beta$. These experimental results are consistent with those by a nonlinear numerical simulation considering a single charged nanochannel, suggesting that parallelization of charged nano-capillaries might be a good upscaling model for a nanoporous membrane for RED applications.

## 1. Introduction

Salinity gradient energy (SGE) is a promising and abundant source of renewable energy harnessed by utilizing the chemical potential difference between freshwater and seawater. SGE can be captured using several technologies [1,2], such as reverse electrodialysis (RED) [3], pressure-retarded osmosis [4], and electric double layer capacitor [5]. Among these methods, RED has been an attractive, pollution-free alternative to harness the Gibbs free energy of mixing of electrolyte solutions [6]. A typical large-scale RED setup consists of a stack of fluidic cells with alternating cation- and anion-exchange membranes, whereby alternating dilute and concentrate salt solutions flow to generate ionic fluxes and, hence, renewable energy output across the electrodes at the two ends of the setup [6]. The RED technology could extract a power amounting to 2.24 MW when 1 m${}^{3}$ of freshwater is mixed with seawater [7], thereby potentially producing a sustainable power output of ≈2.6 TW [8] estimated with a global freshwater flow-rate of ≈1.2 $\times \;10^{6}$ m${}^{3}$/s [9].

The first experimental concept of RED was developed by Pattle (in the 1950s), using alternating chambers of fresh and saline water separated by acidic and basic membranes [10]. However, not until recently have there been active theoretical, numerical, and experimental investigations aiming at pilot projects [11,12], efficient membrane materials [13,14,15,16,17,18,19,20,21,22], improved large-scale cell designs [22,23,24,25,26,27,28], and better understanding of the underlying mechanisms at pore-scale charged interfaces [29,30,31] for optimal power outputs [1,17,32,33]. However, it is challenging to carry out rigorous theoretical and numerical analyses to fully compute and accurately predict the electric outputs of a RED system. The challenges stem from the coupled, nonlinear equations (of ion concentration, electric potential, flow velocity fields) as well as the complex effects of electrokinetics and ion concentration polarization involving charged nanochannels or nanoporous membranes [34,35,36,37].

Rigorous theoretical RED studies usually comprise simplified models of charged ”capillary” membranes, using a single charged nanochannel to represent a typical (ion-exchange) membrane pore [38,39]. With 1D (Poisson-Boltzmann) linearization of ionic concentrations, the analytical results show that the ratio of nanopore or nanochannel radius (*R*) to the Debye length ($\lambda_{D}$) plays a crucial role in determining membrane potential. More specifically, the maximal RED conversion efficiency decreases significantly by increasing the $R/\lambda_{D}$ from 0.1 to 10. These findings critically imply that the size and charges of charged nano-pores/channels and electrolyte concentrations, which affect $\lambda_{D}$, influence the RED efficiency and electric potential generated. On the numerical front, the electrical responses of a single charged nanopore in two-dimensional micro/nano-junctions have been investigated without external flow [40,41], with an external flow of electrolytes [42], and for inhomogeneous surface charge [43]. These studies reveal optimal nanochannel length varying with ion concentrations [40,41], a crucial role of nanochannel height on power output density and ion transfer [42], as well as the dependence of logarithmic current on the ratio of nanochannel length to height [43], respectively.

To elucidate pore-scale observations and understanding, as a bottom-up approach, researchers have recently investigated micro/nano-fluidic RED processes to measure *I*-*V* characteristics of a single straight [44] or an array of charged nanochannels [16,36,45,46,47] between two microfluidic reservoirs under Δc. The studies of conical nanochannels have also attracted much research endeavor recently due to the intriguing nonlinear *I*-*V* response of ionic current rectification for biomedical applications [48,49,50,51]. Directly addressing RED applications, a single-pore (polyimide) membrane system under Δc (using KCl solutions ranging from 1 to 1000 mM) was fabricated that could generate a power output of ≈26 pW and estimated to upscale the power density by ≈1000× with parallelism [29,52,53]. Besides, a membrane-less RED cell design with closely-packed nanoparticles reported a greater power output of the order of magnitude of nW [20,54,55].

To optimize large-scale implementation of RED or blue energy technology using membranes, as a top-down approach, multiple compartment RED cells have been constructed [28,56,57]. Recent studies have shown several crucial influences of ion-exchange membranes [3,58,59] of different materials (e.g., microfiltration [60], polymeric [15,61,62], graphene [18,20], Nafion [13,63]), surface charges, and pore-size distributions, electrolyte solutions (e.g., types of ions [64,65,66], multivalent ions [67], ion concentrations), as well as hydrodynamics affected by the flow configurations [68] and cell designs through separators’ dimensions [23,24,25,26]. Typical power density outputs measured with various ion exchange membrane stacks ranged from 0.13 to 2.48 W m${}^{-2}$ [32].

Because of complex variations of RED components, electrical measurements done with various RED systems could not be comparatively compared with each other directly [69,70]. Moreover, electric measurements and resultant power density conducted with microscopic charged nano-channels or pores can differ significantly from those by the RED stack-membrane systems by several orders of magnitude. This discrepancy implies that a proper upscaling model from microscopic nanochannels to macroscopic RED stacks is missing and remains challenging [32,69,70,71,72]. In this study, we hence developed a convenient mesoscale experimental platform (of a single compartment) to systematically investigate the steady-state electrical characteristics of an ion-exchange membrane under several vital parameters. In particular, we used a commercially available Nafion membrane, which is also commonly used for generating fuel cell renewable energy. We measured the Nafion’s internal resistance, open-circuit, and power output density under different salt concentrations and external flow rates, while the latter influence is hardly explored in the literature. The fluidic platform made with additive manufacturing can be extended to systematically investigate the optimal operating conditions for other membranes under various crucial parameters, e.g., Δc, ion types, external flows, and separation gaps, facilitating systematic electrical measurements and comparisons between different ion-exchange membranes.

## 2. Materials and Methods

**Figure 1:**
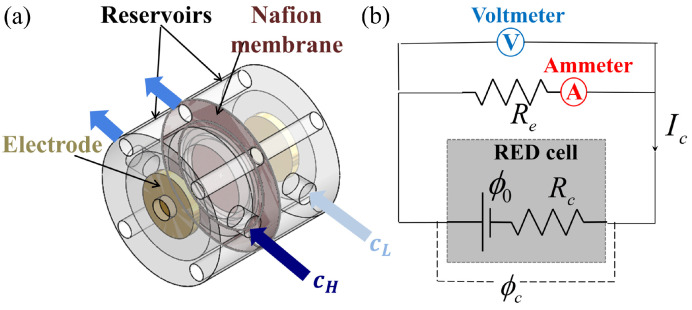
(**a**) Schematic diagram and (**b**) the equivalent electrical circuit of the reverse electrodialysis (RED) cell, comprised of two reservoirs separated by a cation-selective Nafion membrane. Solutions of different salt concentrations ($c_{H}$ and $c_{L}$) flow through the reservoirs. When connecting an external circuit to the two side-electrodes, we obtain the open-circuit voltage ($\phi_{0}$) and internal resistance ($R_{c}$) of the RED cell under a constant flow rate, by measuring the operating voltage ($\phi_{c}$) and current ($I_{c}$) with a voltmeter and ammeter, respectively, while varying the external resistance ($R_{e}$) connected.

Figure 1a illustrates our RED renewable power generation unit, consisting of two reservoirs separated by a cation exchange membrane. A steady-state flow condition of solutions at different concentrations is maintained across the membrane. A concentration difference is hence generated across the membrane and acts as a driving force. The membrane, however, allows only the cations to flow across to the other side, thus creating an ionic flow across the membrane. This ionic flow, in turn, is harnessed using electrodes at the two-side ends of reservoirs. The fluidic cell was cylindrical with a diameter of 40 mm and contained two internal chambers for the passage of high and low concentration solutions, respectively. The diameter and height of each inner cylindrical section were 25 mm and 12.5 mm, respectively. The fluidic cell was fabricated with a 3D printer (Form 2) using stereo-lithography. A syringe pump was used to inject salt solutions of different concentrations through the two reservoirs at a particular flow rate.

**Figure 2:**
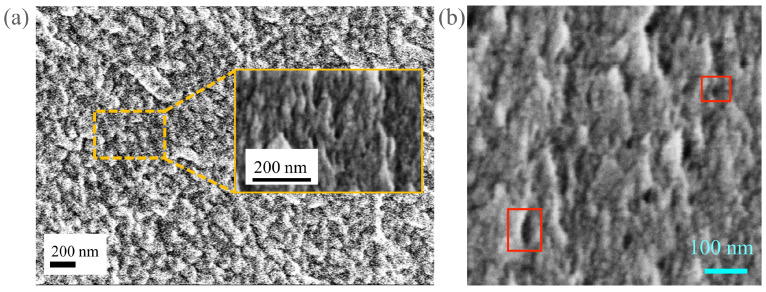
(**a**) Representative SEM image of the Nafion membrane used with approximate nanopore diameter of 20–25 nm. The nanopore size distribution is analyzed with ImageJ image analysis of high-resolution SEM images, such as (**b**). As illustrated, a couple of red boxes in (**b**) enclose typical nanopores, which are many and appear to be black in (**b**).

The salt solution was made by dissolving various amounts of sodium chloride (NaCl) in deionized water. The lowest concentration solution used is deionized water, without any salt added to maximize the electrical output. The cation-exchange membrane (CEM) used is a commercially available Nafion membrane, which is also used for fuel cell research and applications. The membrane has a typical thickness of 127 µm, a conductivity of 0.1 S/cm [73], a tensile modulus of 114 MPa (water-soaked, at 23 ${}^{\circ }$C), the water content of 5%, and a water update of 38% (Nafion${}^{\mathrm{TM}}$ 115, The Fuel Cell Store). The Nafion nanopores varied in shapes and sizes and were observed under a scanning electron microscope (SEM) to find an approximate pore-diameter to be 20–25 nm, as shown in Figure 2. The average pore size is comparable to the Debye length of O(10 nm) with NaCl concentration of 1 mM. The cross-sectional diameter of the membrane held by the fluidic assembly is 17 mm, corresponding to a cross-sectional area of $A_{c}$ = 227 mm${}^{2}$.

When electrolyte solutions with a salt-concentration difference flow across the CEM, the RED cell-assembly (Figure 1a) acts like a miniature battery with an electric potential ($\phi_{0}$) and internal resistance ($R_{c}$), as depicted in Figure 1b. $\phi_{0}$ is known as the open-circuit voltage, i.e., the voltage output when no current flows through the electrical circuit. When connected to an external resistance ($R_{e}$), the voltage supplied by the RED cell is $\phi_{c}$ with an electric current, $I_{c}$. A voltmeter (Keithley 2450 Sourcemeter) and an ammeter (DigiKey 2831E) were used for electrical measurements of $\phi_{c}$ and $I_{c}$, respectively, while a variable resistance was used to control the external resistance, $R_{e}$. We used ten different values of $R_{e}$ between 20 k–700 k and the current ($I_{c}$) and voltage ($\phi_{c}$) readings were recorded at 15 Hz for one minute. The *I*-*V* characteristics of the RED cell were obtained from the time-averaged values of $I_{c}$ and $\phi_{c}$, reaching a steady-state.

Based on the electrical circuit diagram (see Figure 1b), the *I*-*V* relation can be mathematically described as: $\phi_{c}=\phi_{0}-I_{c}R_{c}$. Using this equation, both $\phi_{0}$ and $R_{c}$ of the single-compartment RED cell can be calculated with a best linear-fit of the *I*-*V* measurements (i.e., $\phi_{c}$ and $I_{c}$ data) from the y-intercept and the slope, respectively. One can also express $\phi_{c}$ using the external resistance ($R_{e}$): $\phi_{c}=I_{c}R_{e}$. Using the above two relations, the RED power output $P_{c}^{\prime }\left(=\phi_{c}I_{c}\right)$ can be obtained via the internal and external resistances via:

(1) $$\begin{array}{c} P_{c}^{\prime }=\phi_{0}^{2}\frac{R_{e}}{{\left(R_{e}+R_{c}\right)}^{2}}. \end{array}$$

By optimization, the RED power output, $P_{c}^{\prime }$, has a maximum when the operating voltage is equal to the half of the open circuit voltage: $\phi_{c}=\phi_{0}/2$, and can be estimated via:

(2) $$\begin{array}{c} P_{c,m}^{\prime }=\phi_{0}^{2}/\left(4R_{c}\right). \end{array}$$

The maximum power output density, $P_{c,m}$, is the maximum power output ($P_{c,m}^{\prime }$) per unit cross-sectional area of the membrane ($A_{c}$). We analyzed the effects of Δc and flow rate on the electrical outputs and power density of the RED cell. We further compared our experimental data with numerical results of the RED electrical characteristics using a single charged nanochannel [42].

## 3. Results and Discussions

### 3.1. Influence of External Flow Rates

**Figure 3:**
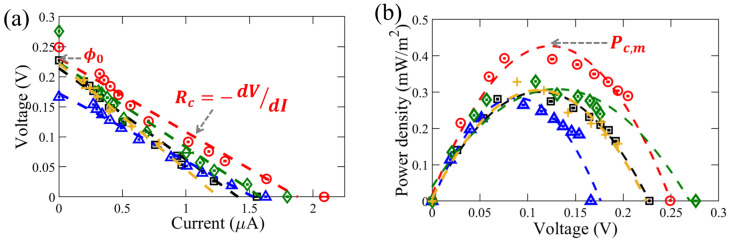
(**a**) Typical measurements of current-voltage (*I*-*V* or $\phi_{c}$-$I_{c}$) and (**b**) the corresponding power density at different voltages of the RED cell using a Nafion membrane. Measurements of the open-circuit voltage ($\phi_{0}$) and membrane internal resistance ($R_{c}=-dV/dI$) are obtained from the y-intercept and slope of the best linear fit of the *I*-*V* curve, respectively. The greatest value of the power density curve in (**b**) denotes the maximum power density, $P_{c,m}$. Here, each set of the experiments are ran at different flow rates, $q_{f}$ = 10 (black square, □), 100 (red circle, ◯), 500 (blue triangle, △), 750 (green diamond, ◊), and 1000 (yellow plus, +) µL/min, but with a constant salt-concentration difference of Δc = 0.86 M. The best linear fitting results of $R_{c}=-dV/dI$ for various $q_{f}$ are shown by the dashed lines in (**a**), with the same color as the corresponding data set. Similarly, the best quadratic fitting results of the power density, $P_{c}=P_{c}^{\prime }/A_{c}=\phi_{c}I_{c}/A_{c}$, varying with $\phi_{c}$ for various $q_{f}$ are shown in (**b**) by the dashed lines (with the matching color as the data set in (**a**)), revealing that the maximum power density ($P_{c,m}$) occurs at $\phi_{c}\approx \phi_{0}/2$.

Revealed in Figure 3a are the typical *I*-*V* measurements obtained for the RED cell under different flow rates, $q_{f}$. In the range of flow rates investigated (10–1000 µL/min), voltage $\phi_{c}$ has an inverse linear dependence on $I_{c}$, showing a constant internal resistance ($R_{c}=-dV/dI$) of the RED cell. The general *I*-*V* trends in Figure 3a reveal a linear decrease of $\phi_{c}$ with increasing $I_{c}$ since $\phi_{c}=\phi_{0}-I_{c}R_{c}$ and both $\phi_{0}$ and $R_{c}$ remain nearly constant for a particular RED setup with specified solution concentrations. Furthermore, as revealed by Figure 3a, the I-V response does not vary significantly with the change in the flow rate of the solutions. The power output density ($P_{c}$) from our setup can be found out from the *I*-*V* data using the relation: $P_{c}=I_{c}\phi_{c}/A_{c}$, where $A_{c}$ is the membrane cross-sectional area confined by the fluidic assembly. Figure 3b shows the results of the power output density, $P_{c}$, for various $q_{f}$. In good agreement with Equation (2), the maximum power output density is always observed when $\phi_{c}=\phi_{0}/2$ for all the cases.

**Figure 4:**
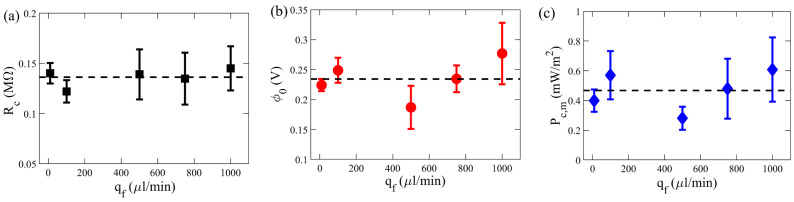
Insignificant variations of (**a**) internal resistance, $R_{c}$, (**b**) open-circuit voltage, $\phi_{0}$, and (**c**) maximum power output density at Δc = 0.86 M under a wide range of flow rate, $q_{f}$. The dashed lines in (**a**–**c**) represent the average value of $R_{c}$, $\phi_{0}$ and $P_{c,m}$, respectively.

We experimentally investigated the influence of flow rates on the RED electrical and power outputs. For each flow rate, at least three experiments were conducted to obtain the error bars reported based on the standard deviation, while keeping the concentration difference constant (Δc = 0.86 M). As illustrated by Figure 4, the internal resistance of RED cell, open-circuit voltage, and maximum power density demonstrated a slight variation with changes in the inflow rate. The average maximum power output density, $P_{c,m}$, was found to be around 0.5 mW/m${}^{2}$. The average values of internal resistance ($R_{c}$) and open-circuit voltage ($\phi_{0}$) were calculated to be 0.14 MΩ and 0.23 V, respectively.

In the experiments, we explored different flow rates over three orders of magnitude, ranging from 10 to 1000 µL/min. We found that a higher flow rate above 1 mL/min is too fast to achieve a complete set of steady-state electrical *I*-*V* measurements for our setup. Therefore, the flow range explored corresponds to a low Peclet number regime, where the ratio of advective to diffusive transport rate is small. Here, we estimate $Pe=v_{f}h/D$, with the flow velocity, $v_{f}$, a characteristic length scale for the RED processes, *h*, which could be approximated with the membrane thickness or dimension of the nanopores, and ion diffusion coefficient, *D*. When approximating *h* with the nanopore dimension or membrane thickness, the corresponding Peclet number ranges between $10^{-9}$ and $10^{-5}$ for our setup, where diffusion is expected to dominate over advection.

### 3.2. Effect of Concentration Difference

**Figure 5:**
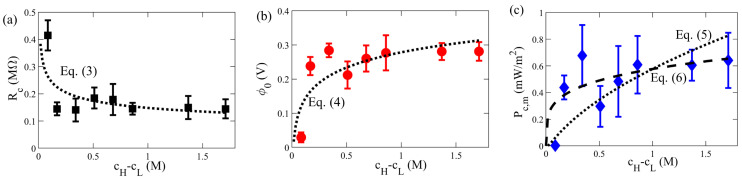
Variation of (**a**) internal resistance, $R_{c}$, (**b**) open-circuit voltage, $\phi_{0}$, and (**c**) maximum power output density, $P_{c,m}$, at $q_{f}=$ 1000 µL/min for various concentration difference, Δc ($=c_{H}-c_{L}$). The dotted lines in (**a**–**c**) represent Equations (4) and (5), respectively. The dashed line in (**c**) demonstrates the curve-fit using the simplified relation (6).

We further carried out a systematic study of a wide range of concentration difference $\Delta c\;(=c_{H}-c_{L})$, ranging from 0.51 mM to 1.71 mM. For the electrical measurements, we kept the salt concentration of the dilute solute fixed ($c_{L}\approx 0$), while the molarity of the concentrated solution was varied. The flow rate was kept constant at 1000 µL/min for studying the effect of Δc. We carried out investigations with five different values of Δc. For each Δc, at least three independent experiments were performed. Figure 5 demonstrates the effect of Δc on the electrical characteristics, namely internal resistance, open-circuit voltage, and maximum power density, of the RED cell using a Nafion membrane.

Shown in Figure 5a is the measurement of nanochannel internal resistance, $R_{c}$, under different Δc. When Δc≳0.2, $R_{c}$ is nearly constant, with little variation with Δc. Besides, we observed a power-law dependence of the internal resistance on the concentration difference. In comparison, an empirical power-law relation between $R_{c}$ and Δc was previously reported, with a 2D numerical simulation for a single charged nanochannel RED cell [42]:

(3) $$\begin{array}{c} R_{c}=A\Delta c^{-\alpha }, \end{array}$$

where *A* and α are constants independent of Δc but could depend on other factors. In the numerical model compared [42], coupled, nonlinear equations, based on the Poisson equation and the conservation of mass, momentum, and charged species (i.e., the continuity, Navier-Stokes, and Nerst-Planck equations, respectively) are simulated to obtain electrical potential, flow velocity, ionic concentrations, and hence the resultant electrical outputs. Although different configurations of charged interfaces are used here, our case of the RED experiment using the Nafion nano-porous membrane shows such a power-law relation of $R_{c}$, given in Equation (3), with the pre-factor 0.15 and the power-law scaling α = 0.23.

The error bars in both Figure 4 and Figure 5 are obtained from 3–5 independent sets of experiments, showing ≈15% and 25% on average, respectively. Although the raw electrical *I*-*V* data such as Figure 3 reveals stable and steady-state values, each independent experiment may still exhibit some reasonable variation due to external flow and curve fittings to obtain the final overall RED outputs.

### 3.3. Comparison with Numerical Results

To explain the above power-law relation observed for our experimental membrane cell, one could approximate the nano-porous CEM membrane as bundle of charged nano-channels parallel to each other. To a first order approximation, for *N* number of parallel nano-channels (each with internal electrical resistance $R_{i}$) the effective electric resistance $R_{\mathrm{eff}}$ could be estimated as *N* numbers of electrical resistors in parallel and, hence, $1/R_{\mathrm{eff}}=\sum_{i}^{N}1/R_{i}$, i.e., $R_{\mathrm{eff}}\approx A\Delta c^{-\alpha }/N$, also exhibiting a power-law relation.

Shown in Figure 5b in our further experimental investigation of the dependence of the open-circuit voltage, $\phi_{0}$, on Δc, revealing a logarithmic dependence. The general trend shows that $\phi_{0}$ is increased with Δc, as a larger Gibbs free energy of mixing is available to harness electricity. Intriguingly, such a logarithmic relation was also reported by the numerical simulation of a single charged nanochannel RED between the two reservoirs, with the following expression [42]:

(4) $$\begin{array}{c} \phi_{0}=B\ln\Delta c+\beta , \end{array}$$

where *B* and β are constants independent of Δc but may depend on other parameters, such as membrane type and geometry, which were not explored in the simulation [42].

Fitting our data with the logarithmic relation of Equation (4), we found the best fitting results of *B* = 0.065 and β = 0.28, based on a least-square residual fitting method. Our experimental data shows that $\phi_{0}$ can be approximated to a constant for a sufficiently large Δc, which is consistent with the logarithmic relation of Equation (4) since $\phi_{0}\approx \beta$ for large Δc.

Figure 5c reveals how the maximum output power density, $P_{c,m}$, varies with Δc. Despite scattering of the data, the general trends can be consistently described by the above empirical relations found for $R_{c}$ and $\phi_{0}$ varying with Δc. Using Equations (3) and (4), one can obtain an empirical relation for $P_{c,m}$ from Equation (2) [42]:

(5) $$\begin{array}{c} P_{c,m}=\frac{\phi_{0}^{2}}{4R_{c}}\frac{1}{A_{c}}=\frac{{\left(B\ln\Delta c+\beta \right)}^{2}}{4A\Delta c^{-\alpha }}\frac{1}{A_{c}}, \end{array}$$

where α is the same theoretical exponent as in Equation (3).

Consistently, our experimental data of $P_{c,m}$ shown in Figure 5c yields the best fitting result of α≈0.26. Considering the approximation $\phi_{0}\approx \beta$, this equation can be further simplified to:

(6) $$\begin{array}{c} P_{c,m}\approx C\Delta c^{\alpha }/A_{c}, \end{array}$$

where *C* (=$\beta^{2}/4A$) = 0.131. The maximum power output density, $P_{c,m}$, was observed to remain in the range of 0.4 to 0.6 mW/m${}^{2}$ with change in Δc between 0.51 and 1.71 mM. In comparison, other experiments have reported a power density, $P_{c,m}\approx$ 0.5 W/m${}^{2}$, using 10 units of commercial ion-exchange membranes, such as Ralex, Neosepta, and Fujifilm (e.g., Figure 4 by Vermaas et al. [67]), and a maximum power output $P_{c,m}^{\prime }\approx 1$ µW (e.g., Figure 10 by Chein et al. [74]) from a single cell RED design using a charged alumina membrane under $c_{H}/c_{L}=1000$ with $c_{L}=10^{-4}$ M. In addition, the typical power density output using a stack of membranes was found in the range of O(0.1–1) W/m${}^{2}$ [32], our single-unit RED results could consistently show such trends when increasing the membrane pairs, decreasing separation distance, and enlarging membrane surface area. The handy lab-scale single compartment fluidics can be extended to explore various complex RED parameters conveniently.

## 4. Conclusions

A RED power generation unit was designed, consisting of two reservoirs separated by a cation-selective Nafion membrane. Solutions of different salt concentrations flow through the reservoirs, establishing a concentration gradient across the nanoporous membrane. This setup generates electricity by exploiting the membrane charge-selectivity and electro-migration of ions by harnessing renewable salinity gradient energy. In the present study, we analyzed the effect of concentration difference and flow rate on the RED electrical *I*-*V* characteristics.

On the one hand, the inflow rate—varied over three orders of magnitudes—shows an insignificant impact on the RED electrical outputs. This may be explained by the dominance of diffusive transport over advection in the low Peclet number nature for the RED cell exploiting nanoporous CEM membrane. On the other hand, the experimental data show that both internal resistance ($R_{c}$) and maximum power density ($P_{c,m}$) demonstrate a power-law dependence on concentration difference, Δc. In contrast, open-circuit voltage ($\phi_{0}$) shows a logarithmic dependence on Δc. Both the power-law and logarithmic relations (of $P_{c,m}$ and $\phi_{0}$ on Δc, respectively) found with the experimental RED using a charged membrane are consistent with previous simulation results of the RED outputs using a charged nanochannel. This implies that parallelization of bundle charged nanochannels could be a good approximation for mimicking an ion-exchange nanoporous membrane as a first-order approximation. The empirical relations of the electrical outputs depending on Δc can be helpful for future designs of similar RED membrane cells.

In terms of applications, experimentally, when Δc≳ 0.2 M, the change in open-circuit voltage with Δc was found to be negligible, implying an optimal operation for the RED membrane cell. Finally, consistent and robust renewable electricity generation with the open-circuit voltage of $\phi_{0}=0.23\;V$ was produced using a Nafion membrane RED cell, with common salt solutions with Δc≳0.2 M.

## Data Availability

Data available upon reasonable request.

## Abbreviations

The following abbreviations are used in this manuscript:

SGE	Salinity gradient energy
RED	reverse electrodialysis
CEM	cation exchange membrane
